# Development of an automated method of detecting stereotyped feeding events in multisensor data from tagged rorqual whales

**DOI:** 10.1002/ece3.2386

**Published:** 2016-09-29

**Authors:** Ann N. Allen, Jeremy A. Goldbogen, Ari S. Friedlaender, John Calambokidis

**Affiliations:** ^1^ Cascadia Research Collective 218 1/2 W. 4th Avenue Olympia Washington 98501; ^2^ Department of Biology Hopkins Marine Station Stanford University Pacific Grove California 93950; ^3^ Department of Fisheries and Wildlife Marine Mammal Institute Hatfield Marine Science Center Oregon State University Newport Oregon 97365

**Keywords:** Accelerometer, archival tag, automated signal recognition, *Balaenoptera physalus*, bio‐logging tag, fin whale, foraging ecology, kinematics, lunge‐feeding, remote observation

## Abstract

The introduction of animal‐borne, multisensor tags has opened up many opportunities for ecological research, making previously inaccessible species and behaviors observable. The advancement of tag technology and the increasingly widespread use of bio‐logging tags are leading to large volumes of sometimes extremely detailed data. With the increasing quantity and duration of tag deployments, a set of tools needs to be developed to aid in facilitating and standardizing the analysis of movement sensor data. Here, we developed an observation‐based decision tree method to detect feeding events in data from multisensor movement tags attached to fin whales *(Balaenoptera physalus*). Fin whales exhibit an energetically costly and kinematically complex foraging behavior called lunge feeding, an intermittent ram filtration mechanism. Using this automated system, we identified feeding lunges in 19 fin whales tagged with multisensor tags, during a total of over 100 h of continuously sampled data. Using movement sensor and hydrophone data, the automated lunge detector correctly identified an average of 92.8% of all lunges, with a false‐positive rate of 9.5%. The strong performance of our automated feeding detector demonstrates an effective, straightforward method of activity identification in animal‐borne movement tag data. Our method employs a detection algorithm that utilizes a hierarchy of simple thresholds based on knowledge of observed features of feeding behavior, a technique that is readily modifiable to fit a variety of species and behaviors. Using automated methods to detect behavioral events in tag records will significantly decrease data analysis time and aid in standardizing analysis methods, crucial objectives with the rapidly increasing quantity and variety of on‐animal tag data. Furthermore, our results have implications for next‐generation tag design, especially long‐term tags that can be outfitted with on‐board processing algorithms that automatically detect kinematic events and transmit ethograms via acoustic or satellite telemetry.

## Introduction

Multisensor, bio‐logging tags are increasingly used to measure the movement, physiology, energetics, and behaviors of animals. This technology has been instrumental in ecological studies, allowing for observation of animals while they are unrestrained and in their natural environment (Cooke et al. 2004). These archival kinematic tags have proven exceptionally useful in environments and time periods where visual observation of behavior is not possible, such as far‐ranging, avian, nocturnal, subterranean, or marine species. Modern movement tags incorporate a variety of sensors including GPS position, accelerometers, magnetometers, underwater pressure sensors, microphones, gyroscopes, etc. (Yoda et al. 1999; Johnson and Tyack 2003; Goldbogen et al. 2013a,b). This suite of sensors allows scientists to discern a tagged animal's behavior remotely, with varying scales of detail from once‐daily location information that provides insights into habitat use of the animal, to detailed information about the animal's 3D movements recorded multiple times a second. This 3D movement sensing has already provided fundamental information about the behaviors of an ever‐expanding variety of species, including penguins, seals, fish, vultures, badgers, pumas, and cheetahs, across a range of environments (Yoda et al. 1999; Shepard et al. 2008; Grünewälder et al. 2012; Nathan et al. 2012; Broell et al. 2013; Gallon et al. 2013; Watanabe and Takahashi 2013; Wang et al. 2015). The types of behaviors that can be discerned range from simple differentiation between “fast” and “slow” movements (Broell et al. 2013) to extremely detailed ethograms designating activities such as paces of travel (e.g., walking, trotting, running, slow and fast swimming), drinking, eating, resting etc. (Watanabe et al. 2005; Moreau et al. 2009). While bio‐logging tags are extremely useful in recording a diverse range of animal movement data, the duration and detail of such studies have thus far been inherently limited by battery life, data storage capacity, and attachment success. However, rapid advances in computer technology, tagging design and materials, and improvements in attachment methods now allow for much longer on‐animal tag durations, a trend that is likely to continue. This increase in remote sampling capability is beneficial in advancing behavioral, physiological, and ecological research, as it minimizes unknown periods of behavior and improves detection of trends through time and across different scales (Cooke et al. 2004). Initially, all processing of on‐animal tag data into meaningful behavioral ethograms was carried out by researchers knowledgeable in the movement, behavior, and kinematics of the study species. However, the rapid increase in the size and quantity of datasets means that the manual processing of hours to weeks of detailed data will quickly become a rate‐limiting step. Development of tools that can leverage the detailed researcher knowledge of the behavior and movements of the study species and use this knowledge to program automatic detection of behavioral events in tag data would greatly reduce analysis time. This would then allow for efficient data processing as well as greatly aid in the standardization of methods and detection rates across studies and species.

A wide variety of automatic behavioral detection methods have been introduced in the literature, with differing strengths and weaknesses to each. In cases where the behavioral states are unknown to the observer, unsupervised machine learning techniques have been used to classify movement data into behaviors (Sakamoto et al. 2009). This method requires almost no prior knowledge of the study species’ behaviors, but alternatively will not discern all behaviors or always classify them correctly (Sakamoto et al. 2009). Machine learning techniques have also been criticized as being “black box” methods that are difficult to implement and with the criteria used in the behavioral selection inaccessible to the user (Bidder et al. 2014). Other studies have utilized various forms of supervised machine learning techniques, in which a training dataset is used to inform the criteria selected by the algorithm (Grünewälder et al. 2012; Nathan et al. 2012; Gao et al. 2013; Bidder et al. 2014). These types of methods are valuable when dealing with multiple types of behaviors, and datasets with high‐quality training data and clearly defined differences in behaviors. However, when classifying a finite number of well‐described behaviors, simple threshold‐based decision rules, or decision tree models, have proven to be very successful (Lagarde et al. 2008; Moreau et al. 2009).

We use the term “decision tree model” to encompass a method used by previous studies under a variety of names. Here, we define it as a series of hierarchical thresholds and criteria that together can be used to classify movement tag data into discrete behaviors. This method has been used in various forms and complexity in many previous studies. Broell et al. (2013) employed a simple threshold detector to find peaks in acceleration signals that indicate a fast versus slow behavioral event in fish tags. Lagarde et al. (2008) found a single acceleration pattern could not distinguish between the behaviors of tortoises, and instead utilized a sequence of movement and the corresponding acceleration values to define the behaviors. For more challenging behavioral identification, Owen et al. (2016) calculated a novel parameter and combined it with a clear series of linear decision rules to identify the timing of feeding events in large whales. All of these methods require a detailed knowledge of the study species, and the use of skilled observers to confirm the automated method behavioral identification (Lagarde et al. 2008; Broell et al. 2013; Owen et al. 2016). At varying levels of complexity, decision tree methods are being used across a wide variety of taxa as a simple, easily implemented method of automated activity detection.

In remotely collected data, the identification of behaviors and activity budgets, particularly those such as feeding, represents biologically significant vital rates, which are extremely relevant to individual and population health, and ultimately the conservation of the species (Shepard et al. 2008). Energetics is a common and important metric in physiological and ecological studies, as foraging rates determine the energy gain and fitness of an individual. Accurate and continuous identification of foraging activity is vital to these energy budgets, as it gives insights into factors such as dive efficiency for marine animals (percent of dive time spent foraging) (Watwood et al. 2006), metabolic rate, productivity, and foraging ecology (Hazen et al. 2009). These are useful for both increased scientific knowledge of a species and identification of changes in foraging behavior and energy budgets in studies of anthropogenic effects on animals.

However, identification of foraging activity from bio‐logging tags has proven a sometimes difficult task. Studies have focused on metrics such as jaw opening, stomach temperature, and head or body acceleration with mixed success (Ancel et al. 1997; Goldbogen et al. 2006; Liebsch et al. 2007; Moreau et al. 2009; Hanuise et al. 2010; Viviant et al. 2010; Kokubun et al. 2011; Grünewälder et al. 2012; Simon et al. 2012; Gallon et al. 2013; Owen et al. 2016). One group of animals that has had proven success in the identification of foraging behavior from tag data are the rorqual whales, which feed by a stereotyped, extremely large kinematic event that is identifiable in tag sensor data (Goldbogen et al. 2006, 2013a,b; Goldbogen 2010; Simon et al. 2012; Owen et al. 2016).

Multisensor tags have been extensively used in the study of the behavior of marine mammals (Johnson and Tyack 2003; Johnson et al. 2009), animals that spend most of their lives below the surface of the ocean where direct observations cannot be made. Baleen whales (*Mysticeti*) are among the largest animals in the world, yet they feed on some of the smallest, such as krill and other zooplankton. By feeding at the bottom of the food web, they are able to reap the benefit of greater biomass available for consumption, which allows them to obtain their large size (Werth 2000). In order to accomplish the task of consuming vastly smaller prey, they employ a method of filter feeding, using large baleen plates to sift through the water for food (Brodie 1977; Pivorunas 1979; Werth 2001, 2013). Rorquals (Balaenopteridae), some species of which represent the largest baleen whales, capture their prey by a method of intermittent filter feeding called lunge feeding (Brodie 1977; Pivorunas 1979; Werth 2001, 2013).

During a lunge, a whale accelerates rapidly toward its prey, opens its mouth to almost 90 degrees, causing flow‐induced pressure that expands the ventral throat pouch around a large volume of prey‐laden water (Orton and Brodie 1987) and causes a rapid deceleration. After the jaws close around the engulfed water mass, the distended ventral pouch is then depressed, forcing the water past the baleen plates to filter the suspended food items (Pivorunas 1979; Kawamura 1980). The rapid acceleration followed by a sudden deceleration gives lunge feeding a kinematic signature that can be easily identified in tag sensor data (Goldbogen et al. 2006).

Until recently, data on lunge‐feeding behavior and kinematics were restricted to limited surface observations (Andrews 1909; Watkins and Schevill 1979; Watkins 1981) or were inferred from anatomical dissections (Goldbogen 2010). However, the advent of high‐resolution archival tags, incorporating movement and sound sensors, now allows observations at depth as well as at the surface (Johnson et al. 2009; Brown et al. 2013). Most studies on lunge‐feeding behavior have focused on deep foraging dives (Goldbogen et al. 2006; Ware et al. 2011; Simon et al. 2012; Friedlaender et al. 2013), and foraging near the sea surface remains poorly understood (Doniol‐Valcroze et al. 2011; Wiley et al. 2011; Goldbogen et al. 2013a,b, 2014). When a movement tag breaches the surface of water, the impact of the air–water boundary causes a large spike in all tag sensors which may affect digital signal processing (i.e., signal‐to‐noise ratio). It is unclear whether surface lunge feeding is similar to deep lunge feeding, and whether the kinematic signatures are equally detectable given the conditional differences.

We developed a decision tree‐based algorithm for automatic detection of lunge‐feeding behavior, both at the surface and at depth, using tag data collected from fin whales, *Balaenoptera physalus*, a large rorqual whale. We identified the sensor metrics that were most successful in recognizing foraging events, the most effective data sampling rates, and quantified the accuracy of the automated detector based on manual observations of the data and prior knowledge of the behavior. The automated feeding detection method outlined here is applicable, with informed modifications, to other feeding rorqual species. It furthermore demonstrates the development of a straightforward automatic behavioral detection method that utilizes behavioral and kinematic knowledge of a species. This type of approach proved extremely successful and potentially provides a template for development and modification to a wide variety of species and behaviors across a range of ecosystems.

## Materials and Methods

This study uses data from suction cup digital acoustic recording tags (DTAG) attached to 19 feeding and nonfeeding fin whales from 2010 to 2013 in waters around the Southern California Bight. The animals were tagged as part of the Southern California Behavioral Response Study (Southall et al. 2012). DTAGs are archival tags that record acoustic data on two hydrophones, and movement data using a pressure sensor and three‐axis accelerometers and magnetometers (Johnson and Tyack 2003). There were four sensor sampling frequencies utilized in this project for the movement sensors: 50, 200, 250, and 500 Hz (Table 1), these sampling rates were used to split the data into low‐frequency (50 Hz; LF) and high‐frequency (200–500 Hz; HF) sampled data. In addition, lunges occurred at all depths, with a split in depth distribution at 30 m, providing a cutoff to distinguish between shallow and deep feeding.

**Table 1:** Tabulated true‐positive (TP) and false‐positive (FP) rates for each whale

Category	Whale	Sampling Rate (Hz)	Shallow		Deep		Total					
			True Pos (%)	False Pos (%)	True Pos (%)	False Pos (%)	True Pos (%)	False Pos (%)				
LF	1	50	75.0	36.8	84.6	0.0	79.3	23.3				
	2	50	100.0	95.8	–	100.0	100.0	96.8				
	3	50	–	100.0	100.0	0.0	100.0	1.4				
	4	50	–	–	89.1	0.0	89.1	0.0				
	5	50	–	100.0	–	–	–	100.0		**Adjusted**		
	6	50	75.0	48.8	94.1	5.9	82.2	36.2		**True Pos (%)**	**FP (%)**	**Full FP (%)**
	7	50	95.5	23.6	100.0	0.0	95.6	23.2	**Average**	92.1	24.3	36.6
	8	50	–	100.0	98.3	8.0	98.3	11.8	**St Dev**	8.6	33.1	40.0
HF	9	200	–	100.0	–	100.0	–	100.0				
	10	200	–	–	–	–	–	–				
	11	200	–	–	95.2	7.0	95.2	7.0				
	12	500	–	–	100.0	7.6	100.0	7.6				
	13	500	100.0	47.6	–	–	100.0	47.6				
	14	500	92.9	9.2	–	–	92.9	9.2				
	15	200	90.8	13.2	–	–	90.8	13.2				
	16	500	97.8	10.2	–	–	97.8	10.2		**Adjusted**		
	17	500	71.4	37.5	–	–	71.4	37.5		**True Pos (%)**	**FP (%)**	**Full FP (%)**
	18	250	88.2	15.1	–	100.0	88.2	16.7	**Average**	92.3	17.8	26.0
	19	500	100.0	0.0	93.2	12.8	94.2	11.0	**St Dev**	8.8	14.6	29.4
			**Totals**									
			**True Pos (%)**	**Adjusted FP (%)**	**True Pos (%)**	**Adjusted FP (%)**	**True Pos (%)**	**Adjusted FP (%)**				
		**Average**	89.7	30.7	94.9	4.6	92.2	22.0				
		**St Dev**	10.4	27.1	5.4	4.7	8.4	24.0				
				**Full FP (%)**		**Full FP (%)**		**Full FP (%)**				
		**Average**		49.2		28.4		30.7				
		**St Dev**		39.1		43.3		33.8				

Averages for separated LF, HF, shallow, deep, and overall totals are displayed. The FP rates are represented both as an adjusted rate, with 100% FPs removed, and as the full FP rate.

Several variables were derived from the collected DTAG data that were used to help define the kinematic patterns of feeding. In previous studies, LF flow noise from tag hydrophones has been used as an estimate of the speed of the tagged animal (Miller et al. 2004; Goldbogen et al. 2006, 2008; Simon et al. 2012). In this study, the LF flow noise was calculated as the root‐mean‐square sound pressure in the 66–94 Hz band, measured five times a second (5 Hz). The flow noise was then used as a proxy for the relative speed of each animal throughout the deployment, with no correction derived from kinematic factors to adjust to actual speed estimates.

The acceleration rate, or jerk, was also calculated for each whale's tagging record. The jerk was computed as the norm of the difference of successive accelerometer samples for all three axes of the accelerometer (Miller et al. 2004; Simon et al. 2012; Ydesen et al. 2014). The resulting value is the normalized jerk between consecutive acceleration samples and is given in units of m/s^3^. Jerk is a useful indicator of fast movements of a tagged whale because it expresses the rapid changes in orientation and acceleration while removing the slowly changing mean orientation.

**Figure 1:**
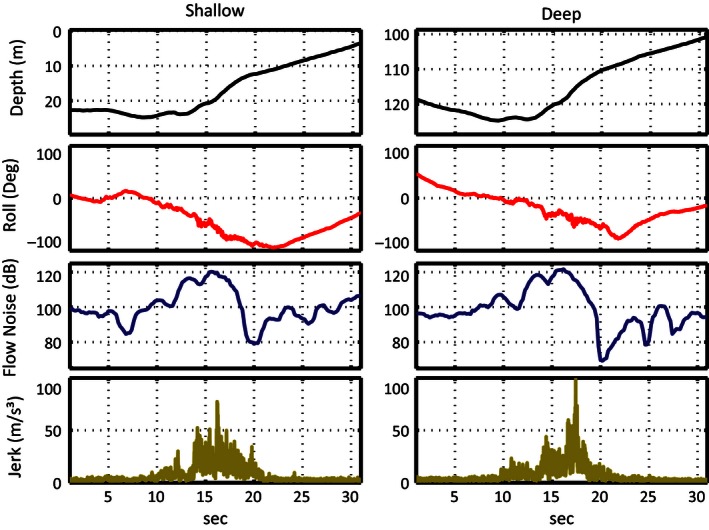
The depth, roll, flow noise, and jerk of a shallow and deep lunge from a whale tagged with a low‐frequency sampled tag.

**Figure 2:**
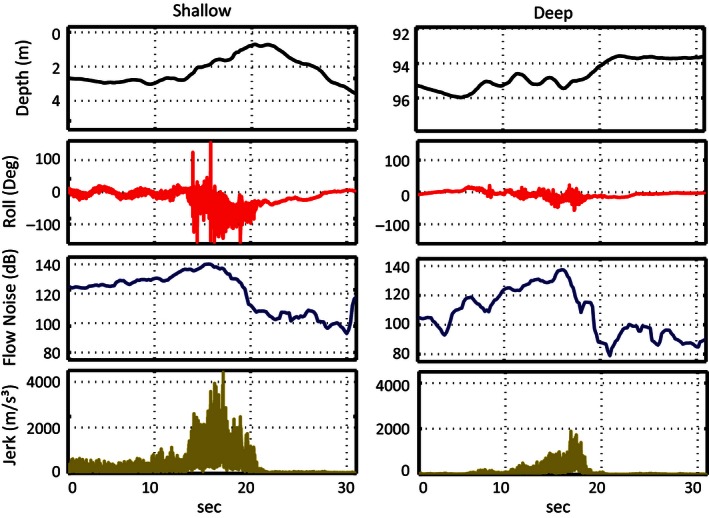
The depth, roll, flow noise, and jerk of a shallow and deep lunge from a whale tagged with a high‐frequency sampled tag.

Using depth (meters), roll (degrees), and jerk, taken at each tag's sampling rate, in addition to flow noise, each whale's tag record was manually examined and the time of each lunge marked. Although there was no direct visual confirmation of lunges, either at depth or the surface, previous studies have inferred the presence of lunge feeding in multisensor tag data based on the known kinematics of how lunge feeding occurs in rorquals (Goldbogen et al. 2006; Simon et al. 2012; Owen et al. 2016). The rapid acceleration of a whale toward a prey patch, and then deceleration as it opens its jaws, is represented in the tag data as an increase and then subsequent drop in flow noise, a sharp spike and then drop in the jerk signal, and often a significant roll (Goldbogen et al. 2006) (Figs. 1, 2). While only deep feeding events have previously been quantified in fin whale tag data, a very similar lunge pattern was observed in surface periods of tag data, indicating feeding activity (Figs. 1, 2).

Once the times were marked for all of the lunges identified in each whale's tag record, an automated detector was designed in MATLAB (MATLAB Release R2013b; The MathWorks, Inc. Natick, MA) using the three variables (flow noise, jerk, and roll) and the observed features of lunging to automatically identify lunge events (detector to be obtained from the authors upon request). A decision tree method was utilized in this study, with thresholds defined based on detailed knowledge of the species and behavior (Goldbogen et al. 2006). More complex techniques, such as fast Fourier transforms (Watanabe et al. 2005) and machine learning methods (Grünewälder et al. 2012; Nathan et al. 2012; Gao et al. 2013), have been utilized to identify behaviors, but other studies have found that simple rules can define expected locomotion patterns within and across species (Lagarde et al. 2008; Shepard et al. 2008; Moreau et al. 2009; Owen et al. 2016).

The lunge times identified by the program were considered correct if they were within 10 sec of a manually selected lunge, and the true‐positive (TP) and false‐positive (FP) detection rates were calculated. The TP rate was calculated as the proportion of automated detections that were correctly identified as lunge events, as defined by the manually selected lunges. The FP rate was calculated as the proportion of automated detections that were incorrectly identified as lunges when no feeding activity occurred. In comparing the detection rates of each method, a preference was placed on reducing FPs over increasing TPs, as incorrectly identifying an animal as feeding when there is no feeding present was deemed less desirable than missing individual feeding events. When feeding rates are utilized in energetic or behavior analysis, the data are often grouped into “feeding” versus “nonfeeding” periods. While missing several lunges during a feeding period will not change this grouping, and will likely have little effect on any energetic rate calculations, a misidentified lunge could misplace an entire dive or time period into a feeding category. This would have a larger affect on the results than a slightly reduced feeding rate.

Multiple combinations of detection thresholds were tested for both shallow and deep lunges, and the TP and FP rates used to compare their efficacy. Based on these repeated tests, an optimized automated detector was designed that identifies both shallow and deep feeding activity.

### Detector design

The utilized method of lunge detection was very similar for both deep and shallow lunging behaviors, with several extra exclusion criteria applied to shallow lunging to reduce additional FPs due to interaction of the tag with the surface. In both methods, the data were binned into one‐second intervals and the mode of jerk, flow noise, and roll, as well as the standard deviation (SD) of roll were taken for each interval. Then, a simple peak detector was used to detect all time bins with the mode of flow noise and jerk, or SD of roll, above a chosen percentage of the rest of the data, 22% for deep lunges and 16% for shallow lunges. Large whales cannot be restrained during tag deployment, so tag placement is often inconsistent, and the farther the placement is away from the body site of the desired activity (in this case the head) the lower the detection signal will be in the data. Therefore, a detection percentage was used instead of an absolute threshold in order to detect peaks in the data.

For continuous sequences of peaks, the maximum value in the sequence was chosen as the actual peak. For shallow lunges, the maximums of the jerk and flow noise that had sequences of less than 2 sec were removed. The bottom percentages of jerk mode were then selected with a threshold of 32% for shallow data and 66% for deep data. The first time bin of these sequences was chosen as the time for a drop in jerk.

A process of elimination was applied to every detected peak for each variable to determine which ones represented lunging activity (Table 2). There was generally a greater roll observed in shallow feeding; therefore, at every selected maximum peak in roll SD in shallow detections, the program checked for three or more consecutive instances of roll mode exceeding 20° in the 3 sec on either side of the roll SD peak. All roll SD peaks that did not meet this criterion were removed from the detections. The time cues of flow noise and jerk peaks were compared, and those that were within 5 sec of each other were kept. These chosen time cues were then compared to the roll SD peaks, and those within 10 sec of each other were kept, as roll tends to be more offset from the acceleration peak. The peaks in flow noise were then tested for a subsequent drop that occurs 8–12 sec after the peak and indicate the opening of the mouth and expansion of the buccal pouch. A drop of 15 dB in flow noise during deep feeding events, and 10 dB drop in shallow feeding, was necessary to consider the detection of a possible lunge.

**Table 2:** Decision table describing the program selection criteria that were used to automatically define feeding activity

**Deep and shallow**
**Shallow**
Jerk, flow noise, SD roll peak detector Shallow: 16% hi, 32% lo; Deep: 22% hi, 66% lo
Three seconds or more of roll mode >20° within 3 sec of SD roll peak
Flow noise and jerk peaks ≤5 sec apart
Flow noise and SD roll peaks ≤10 sec apart
Flow noise drop within 8–12 seconds after peak Shallow: 10 dB; Deep: 15 dB drop
Jerk minimum ≤15 sec after jerk peak
No Jerk peak within ±4 sec of minimum
If >1 detection in 20 sec remove all but final

Dark gray criteria were used to define all lunges, while light gray were used only for shallow data.

These selected detections were then tested for a minimum jerk signal within 15 sec of the peak. During surfacings, the tag breaking the surface of the water leads to spikes in the jerk data that were often chosen by the peak finders. In order to avoid FPs caused by these surfacings, all jerk minimums that also had a jerk peak within ±4 sec were removed, as these would indicate a drop swifter than that seen during lunge feeding.

Lunge‐feeding whales require a minimum amount of time for processing the engulfed water after a lunge before they are capable of lunging again. Goldbogen et al. (2006) found an interlunge interval of 44.5 ± 19.1 sec between speed maxima in feeding fin whales. Here, any detections that were within 15 sec of each other were considered duplicates, and only the last detection kept. The peak detections that fulfilled all of these criteria for shallow and deep data were considered lunges, and the time of the peak in flow noise was taken as the lunge time.

In this study, we attempted to verify the performance of the automated detector against the presence of true lunges as accurately as possible. Manual lunge detecting can be problematic due to highly variable signal types, noise, and combinations as well as human variables such as cognitive load, attention, and fatigue. In order to reduce the variables present under human detection, we verified manual auditing against automated detection to identify both obvious “misses” and more ambivalent events. In cases where a reviewer was unable to confidently discount a lunge, we counted the event as a TP. This practice limits the variable of human error by codifying objective criteria and improving identification fidelity. This objective application of impartial criteria is one of the advantages of automated event detection. In cases of questionable events, an automated method applies the same criteria in every case, whereas a human auditor often employs an unavoidable shifting baseline of comparison as they scan through the data.

## Results

The designed lunge detector was able to identify lunges both at depth and near the surface. The performance of the lunge detector is reported in Table 1. The combined HF and LF data for both shallow and deep lunges had a total TP rate ranging from 71.4 to 100% accurate detection of lunges. The FP rate for all combined lunges ranged from 0 to 100%. There were two 100% FP rates (Whale 5 and Whale 9; Table 1), which considerably skew the detection rates when they are averaged across animals. These 100% FP rates are in animals with no feeding in the entire tag record. This means that even a single FP will produce a rate of 100%. When looking at the actual numbers of detected lunges in these nonfeeding whales, there are only 5 and 7 lunges detected in each of the tag records (Table 3). This is a relatively low number of false detections for multiple hours of tag data. There is a third animal (Whale 10) with no lunges, for which the program correctly recognizes the lack of feeding. Therefore, it is still possible for the lunge detector to perform accurately when there are no feeding events in the data. In order to more correctly represent the performance of the detector, these two FP rates are removed and an adjusted rate presented (Table 1). For the rest of the manuscript, all FP rates are reported as an adjusted average; however, all full FP averages are reported in Table 1.

**Table 3:** The absolute numbers of manually detected, automatically detected, and correctly automatically detected lunges

Category	Whale	Sampling rate (Hz)	Shallow			Deep			Total lunges		
			Lunges	Detected lunges	Correctly detected lunges	Lunges	Detected lunges	Correctly detected lunges	Lunges	Detected lunges	Correctly detected lunges
LF	1	50	16	19	12	13	11	11	29	30	23
	2	50	1	24	1	0	7	0	1	31	1
	3	50	0	1	0	68	68	68	68	69	68
	4	50	0	0	0	46	41	41	46	41	41
	5	50	0	5	0	0	0	0	0	5	0
	6	50	28	41	21	17	17	16	45	58	37
	7	50	44	55	42	1	1	1	45	56	43
	8	50	0	8	0	175	187	172	175	195	172
HF	9	200	0	3	0	0	4	0	0	7	0
	10	200	0	0	0	0	0	0	0	0	0
	11	200	0	0	0	42	43	40	42	43	40
	12	500	0	0	0	73	79	73	73	79	73
	13	500	11	21	11	0	0	0	11	21	11
	14	500	85	87	79	0	0	0	85	87	79
	15	200	65	68	59	0	0	0	65	68	59
	16	500	45	49	44	0	0	0	45	49	44
	17	500	7	8	5	0	0	0	7	8	5
	18	250	51	53	45	0	1	0	51	54	45
	19	500	13	13	13	73	78	68	86	91	81

The totals are shown for shallow and deep feeding, as well as the entire tag record of each animal.

There was a split of tag sampling frequencies, with 8 LF and 11 HF tag deployments. The total TP rate of the LF tags averaged 92.1% with an SD of 8.6 and 92.3% with an SD of 8.8% for HF tags, extremely similar performances. However, when the adjusted FP rates are compared, the LF tags average 24.3% accuracy with an SD of 33.1%, while the HF tags average 17.8% with an SD of 14.6% (Table 1).

When looking at the absolute numbers of false detections, the LF tags have larger variability in inaccurately identified lunges, as well as a larger absolute numbers of false detections, with three animals (Whales 2, 6, 7, and 8; Table 3) having 13 or more false detections, while the HF data have a maximum of 10 FPs per whale (Whale 13 and 19; Table 3). These differences illustrate the greater variability in the performance of the detector for LF tags.

When the lunge detections are separated into shallow and deep detections, there are 11 shallow lunging animals and nine deep lunging animals, with four animals feeding both shallow and deep. Except for Whale 2, which had 24 shallow detections, all animals with no shallow lunges had low (one to eight detections) or no false detections (one LF whale three HF whales) (Table 3). With the removal of animals with no shallow lunges, the TP detection rates are 89.7 ± 10.4% with an adjusted FP of 30.7 ± 27.1%, with the HF tags giving the best shallow lunging detector performance.

Ten animals had no deep feeding activity in their tag records. The detector incorrectly identified lunges in only three of these, with an absolute number of false detections ranging from one to seven (Table 3). For the remaining seven whales, the detector accurately identified the lack of feeding activity. With the nonfeeding animals removed, the TP detection rate is 94.9 ± 5.4% with a FP rate of 4.6 ± 4.7% (Table 1). While the deep and shallow detectors have relatively similar TP rates, the discrepancies in the FPs highlight the added difficulty of lunge detection near the surface.

When the manually selected data were secondarily reviewed against the automated detector, there was a ubiquitous increase in TP and decrease in FP rates, with an overall TP rate of 92.8 ± 6.5% and a FP rate of 9.5 ± 12.1% (Table 4). While the increase in TP rate was modest, there was a sharp decrease in the FP rate as lunges that were ambiguous, often due to a low signal‐to‐noise ratio, were marked correct. This effect was most dramatic in shallow lunging activity (Table 4). The largest of these differences was seen in the LF detector performance, with most of the difference driven by Whale 2, with a decrease in the FP rate from 96.8 to 38.7%. Previously there was only one detected lunge in the entire record (Table 5). This number was greatly increased with secondary evaluation, when the reviewer could not conclusively determine whether the signals represented feeding activity due to high data noise.

**Table 4:** The tabulated true‐positive and false‐positive rates for each whale adjusted for the secondary manual verification of lunges

Category	Whale	Sampling Rate	Shallow		Deep		Total					
			True Pos (%)	False Pos (%)	True Pos (%)	False Pos (%)	True Pos (%)	False Pos (%)				
**LF**	1	50	82.6	0.0	84.6	0.0	83.3	0.0				
	2	50	100.0	45.8	100.0	14.3	100.0	38.7				
	3	50	–	100.0	100.0	0.0	100.0	1.4				
	4	50	–	–	89.1	0.0	89.1	0.0		**Adjusted**		
	5	50	–	100.0	–	–	–	100.0		**True Pos (%)**	**FP (%)**	**Full FP (%)**
	6	50	81.1	26.8	94.1	5.9	85.2	20.7	**Average**	93.1	11.9	22.9
	7	50	95.7	18.2	100.0	0.0	95.8	17.9	**St Dev**	7.1	14.6	33.9
	8	50	–	100.0	98.4	0.5	98.4	4.6				
**HF**	9	200	–	100.0	–	100.0	–	100.0				
	10	200	–	–	–	–	–	–				
	11	200	–	–	95.3	4.7	95.3	4.7				
	12	500	–	–	100.0	5.1	100.0	5.1				
	13	500	93.3	33.3	–	–	93.3	33.3				
	14	500	93.4	2.3	–	–	93.4	2.3				
	15	200	91.4	5.9	–	–	91.4	5.9				
	16	500	98.0	0.0	–	–	98.0	0.0		**Adjusted**		
	17	500	77.8	12.5	–	–	77.8	12.5		**True Pos (%)**	**FP (%)**	**Full FP (%)**
	18	250	89.3	5.7	100.0	0.0	89.5	5.6	**Average**	93.2	7.7	16.9
	19	500	100.0	0.0	94.0	0.0	94.8	0.0	**St Dev**	6.3	10.3	30.8
			**Totals**									
			**True Pos (%)**	**Adjusted FP (%)**	**True Pos (%)**	**Adjusted FP (%)**	**True Pos (%)**	**Adjusted FP (%)**				
		**Average**	91.2	13.7	96.0	3.4	92.8	9.5				
		**St Dev**	7.7	15.6	5.2	4.5	6.5	12.1				
				**Full FP (%)**		**Full FP (%)**		**Full FP (%)**				
		**Average**		36.7		10.9		19.6				
		**St Dev**		41.6		28.4		31.4				

Averages for separated LF, HF, shallow, deep, and overall totals are displayed.

**Table 5:** The absolute numbers of manually detected, automatically detected, and correctly automatically detected lunges for the secondary manual verification of lunges

Category	Whale	Sampling rate (Hz)	Shallow			Deep			Total lunges		
			Lunges	Detected lunges	Correctly detected lunges	Lunges	Detected lunges	Correctly detected lunges	Lunges	Detected lunges	Correctly detected lunges
LF	1	50	23	19	19	13	11	11	36	30	30
	2	50	13	24	13	6	7	6	20	31	19
	3	50	0	1	0	68	68	68	68	69	68
	4	50	0	0	0	46	41	41	46	41	41
	5	50	0	5	0	0	0	0	0	5	0
	6	50	37	41	30	17	17	16	54	58	46
	7	50	47	55	45	1	1	1	48	56	46
	8	50	0	8	0	189	187	186	189	195	186
HF	9	200	0	3	0	0	4	0	0	7	0
	10	200	0	0	0	0	0	0	0	0	0
	11	200	0	0	0	43	43	41	43	43	41
	12	500	0	0	0	75	79	75	75	79	75
	13	500	15	21	14	0	0	0	15	21	14
	14	500	91	87	85	0	0	0	91	87	85
	15	200	70	68	64	0	0	0	70	68	64
	16	500	50	49	49	0	0	0	50	49	49
	17	500	9	8	7	0	0	0	9	8	7
	18	250	56	53	50	1	1	1	57	54	51
	19	500	13	13	13	83	78	78	96	91	91

The totals are shown for shallow and deep feeding, as well as the entire tag record of each animal.

## Discussion

### Method performance

We have demonstrated here an extremely accurate automated feeding detector for fin whales using a decision tree algorithm. The accuracy of the detector does vary with different factors, including depth of feeding and sampling frequency of the tag sensors. There is also variability across individuals in the signal‐to‐noise ratio, which is likely due to differences in tag placement on the animal, as well as physiological or behavioral differences between animals. These are factors that must be overcome regardless of the method used in behavioral detection.

In our study, shallow feeding had a higher FP rate than deep feeding. When an animal approaches the surface, they encounter additional drag forces associated with waves created at the air–sea interface, called wave drag (Hertel 1966), in addition to reduced maneuvering room imposed by the surface barrier. These differences necessitate changes in the kinematics of lunge‐feeding behavior, thus changing the detection of near‐surface feeding. Ambient noise levels also increase with proximity to the surface, with strong wind speed dependence (Urick 1984). This increases the background noise present on the tag hydrophones, making flow noise‐based speed estimates near the surface less accurate as well as decreasing the signal‐to‐noise ratio of the lunge signal. There is further interference of the tag sensors with the surface of the water when the unit breaks the surface, either during breaths or feeding activity. This abrupt density transition, as well as the break in surface tension, creates large spikes in the accelerometer, hydrophone, and other tag sensors, confounding lunge identification which depends on detection of peaks in the data from these sensors.

We have shown here that while they are not as easily or accurately identifiable as deep lunges, fin whale surface feeding is clearly evident in movement sensor data (Figs. 1, 2). Owen et al. (2016) also used a simple set of decision rules to successfully detect surface lunge feeding in DTAG data from humpback whales, another rorqual species. They utilized a novel parameter, excess x‐acceleration, to represent the forward acceleration of the animal regardless of pitch angle and obtained a 70% detection rate of surface lunging (Owen et al. 2016). Their tag accelerometer data were sampled at 50 Hz and then downsampled to 5 Hz during calibration (Owen et al. 2016). In our study, we found that HF sampled sensors reduce the difficulty in identifying surface feeding in tag records with high data variability or noise. In the method we utilize, this difference is almost entirely driven by the only acceleration derived metric, jerk. A higher sampling rate allows for a much higher absolute jerk signal (i.e., higher resolution in changes in acceleration) (Figs. 1, 2). This larger value means that slower acceleration changes from activity such as fluking are not mistaken for lunges in HF tags, providing a much clearer jerk signal and more accurate detector performance.

One of the potential limitations of an automated detector that finds peaks by taking an upper percentage of the data is that it will provide a high number of FPs when there is no feeding present in the data, cueing instead of the next largest signal in the record. HF sampling allows for enough resolving power to greatly reduce this issue. This effect will likely be more pronounced in smaller animals, whose movements are short and quick, and may otherwise be aliased in LF sampled sensors. Accelerometer sampling frequency has been shown to be extremely important for correct behavioral categorization in fish tags, with increased frequencies (>100 Hz) giving the most accurate activity classification (Broell et al. 2013).

The large reduction in the FP rate of Whale 2 likely represents an example of a tag placement or attachment that was not conducive to feeding detection. The program identified a large number of lunges where the manual auditor had originally marked only one. Upon secondary evaluation of the manual detection, the data were ambiguous due to a small signal‐to‐noise ratio, with many spikes in the signals that made lunge identification extremely difficult. These spikes were likely the result of either poor tag orientation or inadequate attachment causing constant jostling of the tag and noise in the sensors. Similar signal noise was often found in the final minutes of a deployment, when the tags began to detach from the animal.

The specific location of movement tags on an animal's body impacts what behaviors can be distinguished from the sensors (Shepard et al. 2008; Gleiss et al. 2010; O'Toole et al. 2011; Brown et al. 2013). In the case of lunge‐feeding rorqual whales, tag placement away from the caudal region will reduce the interference of fluking acceleration signals in lunge detection. It has also been shown that consistent tag placement between individuals greatly improves the signal‐to‐noise ratio and minimizes interpretation errors (Shepard et al. 2008; Brown et al. 2013). While it is difficult to precisely attach bio‐logging tags on large species that cannot be handled during deployment, tag positioning should be carefully considered when studying other species. However, placement issues will make behavioral detection difficult whether it is done manually or by automation.

In this study, we wished to quantify the performance of our automated method by comparing it to the identification of true lunges in the data. There is currently no completely unbiased method of identifying feeding in rorqual species, as there is no way to visually observe the animals during the entirety of the tag deployment. We attempted to reduce human variables such as cognitive load, attention, and fatigue that might otherwise skew the TP detections by secondarily evaluating the manual selection criteria. With no current method of unequivocally determining the presence or absence of lunge activity, we are instead evaluating the ability of the automated method to correctly apply the selection criteria and identify feeding events as accurately as possible. The increased consistency in the application of detection criteria is evident in the overall increase in TP and decrease in FP detection rates across all animals, depths, and sampling frequencies when they were subjected to this secondary evaluation (Tables 1, 4). This improvement in detection rates was due to two factors: uniform application of selection criteria across ambiguous signals when signal‐to‐noise ratio was low, and reduction in the number of “misses” due to human factors.

### Applications and broader context

Using fin whale foraging as a model system, we have developed an extremely effective method of decision tree‐based automated feeding identification in movement tag data. Automation of the classification of movement tag data into activity logs is an extremely important goal in bio‐logging studies for a variety of reasons. There has been a dramatic rise in the absolute number of movement tags deployed, and the number of species they have been used on, with 125 animal species carrying accelerometer tags as of 2013 (Brown et al. 2013). Animal movement tags generate large amounts of data, from hours to days of multiple sensor streams. Development of automated detection methods dramatically decreases the data analysis time necessary to identify behavioral and ecological energy budgets from these types of tag data. Automation of activity recognition also requires standardization of detection methods for each behavioral type which is essential for consistency and cross‐comparability of studies, as well as providing a quantifiable error rate.

In studies of anthropogenic effects on animal populations, identification of vital rates, especially feeding, is extremely important to quantifying human impacts on individuals and populations. Multisensor tags are particularly suited to this type of study, as they allow for remote observations of behavior without direct human influence past the point of deployment. In cases where accurate identification of activity is extremely important, this method can easily be customized to bring up an accuracy check of detected behavioral events. In this way, a human user could examine figures of each marked behavior and determine whether it was correctly identified, further improving the precision of the method. When kinematic tag data are combined with other tag data, such as light levels, temperature, and GPS position, behaviors can be placed in broader ecological contexts (Brown et al. 2013). Expansion of this technique to other behavioral modes such as resting, social interaction, travel, etc. would allow for a more complete classification of the behavioral ecology, and energy budgets of a subject species, and will ultimately greatly improve quantification of the anthropogenic effects impacting it.

Regardless of the method used, automated feeding and behavioral detection opens up the possibility of in situ activity counters in next‐generation, long‐term tag designs, providing real‐time or long‐term ethograms that may more accurately inform researchers of feeding rates or other behaviors that may last beyond the duration of current tags. This would especially benefit species that are most suited to bio‐logging tag studies, those that are remote or inaccessible such as marine, avian, or far‐ranging species. In these cases, the deployment and recovery of the tag is the limiting step. If activity counters can be developed for each species, then behavioral budgets can be calculated on board and remotely transmitted via acoustic or satellite telemetry to researchers. This would increase the practical longevity of tagging records, as it would reduce the importance of recovering deployed tags, given that the data would be preserved in the event of tag loss or destruction.

In our study, we have demonstrated the effectiveness of a simple decision tree‐based algorithm to identify feeding activity in multisensor tag data. Both automated and manual activity detection methods come with advantages and disadvantages. Here, we combine the advantages of both methods, using selection criteria based on observations by a skilled interpreter of expected kinematic patterns, and then applying these criteria in a consistent manner across all signal types and animals in our study. While other more complex methods of detection have been used previously, such as fast Fourier transform (Watanabe et al. 2005), support vector machines (Grünewälder et al. 2012; Nathan et al. 2012; Gao et al. 2013; Bidder et al. 2014), and artificial neural networks (Nathan et al. 2012), many other studies have found that simple rules can successfully define expected behavioral signals in tag data (Lagarde et al. 2008; Shepard et al. 2008; Moreau et al. 2009; Broell et al. 2013; Gallon et al. 2013; Watanabe and Takahashi 2013). Fully or partially supervised machine learning techniques require a high level of computational and programming proficiency and are often performed under “black box” conditions. However, they can be used in a variety of situations with sometimes very little knowledge of the underlying physiology and behavior of the species (Sakamoto et al. 2009).

Decision tree or threshold‐based methods such as we present here require much greater baseline understanding of the study species and behavior. The analysis cannot be performed without a significant prior knowledge of the species, the kinematics of the desired behavior, and a working familiarity with the signatures of the behaviors in the tag data. However, given this knowledge, decision tree methods then employ a basic set of selection rules that define an expected behavior. These set of simple, defined criteria mean they do not require a high level of programming skill and are therefore simple to implement and are readily accessible to a greater number of researchers. In some cases, the data and behavior are straightforward enough that one or two parameter thresholds can be used to answer the study question (Lagarde et al. 2008; Broell et al. 2013). In this study, the interaction of the tag with the surface meant a larger number of decision rules were needed to exclude these surfacing events from the FPs.

Our automated detection algorithm is likely modifiable to other whale species that use the same lunge‐feeding mechanism. Owen et al. (2016) utilized a similar method to the one we present here, providing a further example of simple threshold‐based models that are being actively developed to enhance behavioral detection techniques in marine mammals. A large number of rorqual whales, including fin whales, are endangered species. Accurate assessments of feeding rates and energetic expenditure are essential to the successful maintenance and recovery of these species. Future testing of this method on similar behaviors and species would indicate its relevance to a broader range of important conservation questions.

This method of observation‐based automated activity recognition is also modifiable for quick and accurate detection of any repeated behavior that can be identified by defined thresholds in movement tag sensor data. Bio‐logging tags have already been used to research species in every Earth biome. Miniaturization, as well as increased sampling, battery, and storage capacity, will add to the number of species for which movement tag studies can be utilized. Our methods of activity detection provide a template that other researchers may use for simple, observation‐based automated behavioral detection in all species, terrestrial and avian, in addition to marine, with implications for expanded uses in future studies.

## Conflict of Interest

None declared.
